# ACTH- and Cortisol-Associated Neutrophil Modulation in Coronary Artery Disease Patients Undergoing Stent Implantation

**DOI:** 10.1371/journal.pone.0071902

**Published:** 2013-08-14

**Authors:** Margit Keresztes, Tamás Horváth, Imre Ocsovszki, Imre Földesi, Gyöngyi Serfőző, Krisztina Boda, Imre Ungi

**Affiliations:** 1 Department of Biochemistry, Medical Faculty, University of Szeged, Szeged, Hungary; 2 Invasive Cardiology Unit, Centre of Cardiology, Medical Faculty, University of Szeged, Szeged, Hungary; 3 1st Department of Internal Medicine, Medical Faculty, University of Szeged, Szeged, Hungary; 4 Department of Medical Physics and Informatics, Medical Faculty, University of Szeged, Szeged, Hungary; University of Leicester, United Kingdom

## Abstract

**Background:**

Psychosocial stress and activation of neutrophil granulocytes are increasingly recognized as major risk factors of coronary artery disease (CAD), but the possible relationship of these two factors in CAD patients is largely unexplored. Activation of neutrophils was reported to be associated with stenting; however, the issue of neutrophil state in connection with percutaneous coronary intervention (PCI) is incompletely understood from the aspect of stress and its hypothalamic-pituitary-adrenal axis (HPA) background. Thus, we aimed to study cortisol- and ACTH-associated changes in granulocyte activation in patients undergoing PCI.

**Methodology/Principal Findings:**

Blood samples of 21 stable angina pectoris (SAP) and 20 acute coronary syndrome (ACS) patients were collected directly before (pre-PCI), after (post-PCI) and on the following day of PCI (1d-PCI). Granulocyte surface L-selectin, CD15 and (neutrophil-specific) lactoferrin were analysed by flow cytometry. Plasma cortisol, ACTH, and lactoferrin, IL-6 were also assayed. In both groups, pre- and post-PCI ratios of lactoferrin-bearing neutrophils were relatively high, these percentages decreased substantially next day; similarly, 1d-PCI plasma lactoferrin was about half of the post-PCI value (all *p*≤0.0001). Post-PCI ACTH was reduced markedly next day, especially in ACS group (SAP: *p*<0.01, ACS: *p*≤0.0001). In ACS, elevated pre-PCI cortisol decreased considerably a day after stenting (*p*<0.01); in pre-PCI samples, cortisol correlated with plasma lactoferrin (*r*∼0.5, *p*<0.05). In 1d-PCI samples of both groups, ACTH showed negative associations with the ratio of lactoferrin-bearing neutrophils (SAP: *r* = −0.601, *p*<0.005; ACS: *r* = −0.541, *p*<0.05) and with plasma lactoferrin (SAP: *r = *−0.435, *p*<0.05; ACS: *r = *−0.609, *p*<0.005).

**Conclusions/Significance:**

Pre- and post-PCI states were associated with increased percentage of activated/degranulated neutrophils indicated by elevated lactoferrin parameters, the 1d-PCI declines of which were associated with plasma ACTH in both groups. The correlation of plasma cortisol with plasma lactoferrin in the extremely stressed ACS before stenting, however, suggests an association of cortisol with neutrophil activation.

## Introduction

Although coronary artery disease (CAD) and heart attack have been taught at medical schools for centuries, our comprehension of atherosclerosis as a disease of vascular inflammation is relatively new, and the underlying pathophysiological-pathobiochemical mechanisms are still not fully understood. In the initiation of this inflammatory reaction by oxidized LDL (a major, primary pro-atherogenic factor), mostly monocytes and macrophages are thought to have a primary role - in addition to endothelial cells, while dendritic cells and T cells are supposed to act mainly in subsequent steps [1]–[3]. Recently, however, the unexpected importance of neutrophil granulocytes in the onset and progression of atherosclerosis has come to the limelight [4]–[7]. Surprisingly, it was found that neutrophil count could be associated with the incidence of acute coronary events or may be an independent predictor of multiple stenosis in chronic stable angina [8]–[9]. Reports on the state of neutrophils in stable angina pectoris (SAP) are controversial [4], [10], while systemic neutrophil activation is a consistent finding in unstable angina [11], [12]. Interestingly, it was shown that granulocytes are already primed in hyperlipidemic patients, thus, any second stimulus may trigger full activation of these leukocytes [13].

There is an increasing evidence, that psychosocial stress could play a major part in the development of coronary artery disease. The great INTERHEART study revealed that psychosocial stress is to be regarded as a main risk factor for myocardial infarction [14], [15]. Furthermore, patients with acute coronary syndrome (ACS) are usually extremely stressed, and stable CAD patients may also feel tension e.g. before-during PCI [16]. Thus, it could be most important, that psychological stress itself could result in activation of neutrophils in anxious patients and even in healthy individuals [17]–[19].

In neuroendocrine-immune regulation of the stress response/state, HPA axis has an outstanding role via CRH, ACTH and cortisol [20]. In addition to the well-known role of cytokine/chemokine receptors, neutrophil behaviour and homeostasis are regulated also by stress hormones, e.g. by glucocorticoids via specific receptors [5], [7], [21]. Although cortisol and its analogs are known and widely used as anti-inflammatory agents and immune suppressors, (morning) plasma cortisol levels were reported to correlate with the severity of coronary atherosclerosis in US Air Force aircrew members and in patients with suspected CAD [22]–[23]. Furthermore, cortisol stress reactivity was found to be associated with coronary artery calcification in healthy men and women [24].

Cytokines are suggested to have a central importance both in stress/depression, in atherosclerosis and in CAD; particularly, the role of IL-6 is emphasized as a main regulator of the systemic inflammatory response and as a most potent stimulator of the HPA axis [20], [25], [26]. Stenting/PCI was reported to trigger rapid, transient neutrophil activation, followed by the release of IL-6 and IL-8 in unstable angina; a similar post-PCI elevation of cytokines was reported also in stable angina [27], [28].

So far, the issue of neutrophil activation state in connection with PCI is largely unexplored from the aspect of stress and its HPA background. Therefore, we set out to investigate the state of granulocytes in patients with SAP or ACS - before, directly after and on the next day of PCI; and we wished to examine the possible associations between granulocyte state and plasma ACTH or cortisol. Our results suggest an association between plasma cortisol and a neutrophil degranulation marker (in plasma) in the grossly stressed ACS patients before stenting, while the decrease in the percentage of activated neutrophils was associated with plasma ACTH in both patient groups on the next day of PCI.

## Materials and Methods

### Patients

25 SAP and 20 ACS patients were enrolled for our prospective study. Patients (over 18 years) were eligible if they had characteristic symptoms and findings of either stable angina (with positive stress test) or acute coronary syndrome. (In the ACS group, 6 patients had unstable angina, and 14 patients were diagnosed to have acute myocardial infarction/with increased cardiac troponin T level/; from the latter group, 6 subjects had ECG with ST elevation, while no ST elevations were seen in the ECG of 5 patients.).

For inclusion, at least one significant stenosis (diameter >50%) was to be demonstrated by coronary angiography, as suggested by Videm et al. [10]; angiography was followed by stenting in a single vessel. (In both groups, the patients were included if the PCI could be performed between 8 a.m. –2 p.m., as the flow cytometry procedure was to be finished in the same afternoon.).

Exclusion criteria included prior bypass surgery, cardiogenic shock, malignancy, any immunological disorders or acute inflammations, immunosuppressive or anti-inflammatory treatment (except low-dose aspirin), recent major trauma/surgery, drug/alcohol abuse, poor mental function. Four stable patients’ samples could not be processed (blood clotting disturbances and sample management problems); thus, our study involved 41 patients (27/14 males/females; age 33–87 years). All patients took aspirin in low doses during the study; statins, antihypertensives, sedatives were not discontinued. Clopidogrel (600 mg loading dose, 75–150 mg maintenance dose) was given before and after PCI; all patients were administered heparin (70–100 IU/kg) during PCI.

The study protocol was approved in advance by the Regional Medical Ethics Committee (Human Investigation Review Board, University of Szeged, Albert Szent-Györgyi Clinical Centre; Ref. No: 37/2005). Most participants gave full written consent to participate before enrolling, and after giving detailed informations on the study from the aspect of participants (interactive talk, written study description). In serious acute cases, the patients gave informed written consent after the emergency life-saving intervention was finished. The investigation conformed to the principles of the Declaration of Helsinki.

### Blood Sampling and Laboratory Methods

Blood samples were collected immediately before PCI (pre-PCI), immediately after PCI (post-PCI), and on the following day (1d-PCI). (The mean time of PCI was about 45 min, with a range between 30 min and one hour.) The 1d-PCI samples were collected routinely between 8–9 h a.m. in each case. Blood collection for routine blood analysis (total leukocyte count, neutrophil fraction and lipid panel) was performed at admission for acute patients, and before admission for the stable angina pectoris group (to check exclusion criteria like acute inflammation). CK values were assayed in 1d-PCI samples, in order to monitor (diagnose) heart attack in the ACS group, and to check the occurrance of acute procedural myocardial infarction (a possible consequence of PCI) in the SAP patients.

Blood cell counts, total cholesterol, triglycerides, HDL-cholesterol and CK activity were measured by standard protocols in automatic cell counter/analyzers (Roche); LDL-cholesterol was calculated (Friedewald formula). Reference ranges: total leukocyte count: 3.9–11.1 10^9^/l, neutrophil fraction: 44.0–68.0%, total cholesterol: <5.2 mmol/l, triglycerides: <2.0 mmol/l, HDL-cholesterol: >1.0 mmol/l, LDL-cholesterol: <3.0 mmol/l, CK activity: <200 U/l. Cardiac troponin T assay was performed by a qualitative, rapid dry chemistry test (Roche) using heparinized whole blood, and it was evaluated visually.

ACTH, cortisol, lactoferrin and IL-6 were assayed in EDTA-plasma, plasma aliquots were stored at −80°C for these analyses. ACTH concentrations were determined by a chemiluminescent immunoassay (LKAC1, Immulite 1000; Siemens Healthcare Diagnostics, UK); reference range: 0–60 pg/ml. Cortisol was assayed by radioimmunoassay (DSL-2100; Diagnostic Systems Laboratories, Webster, TX); reference range: 160–620 nmol/l. Plasma lactoferrin was determined by an „in-house” ELISA kit, developed according to Antonsen [29]. Briefly, microplates were coated overnight with rabbit anti-human lactoferrin (3.7 mg/l; Dako, Denmark) at 4°C. After washing twice, plasma samples were applied in duplicates (in wells: ten-fold dilution), and incubated for 1 h at room temperature. Incubation with anti-lactoferrin-peroxidase was carried out similarly, after five washing steps. The conjugate was prepared from Dako antibody and from horseradish peroxidase (Calbiochem, USA) according to Wilson and Nakane [30]; it was used in 4000-fold diluted form. Following washing steps, plates were developed with TMB (BD Biosciences, USA), and reaction was stopped by 4 N sulfuric acid. Human lactoferrin (Sigma, USA) served as standard; reference range/median for plasma lactoferrin: 40–200/90 ng/ml [29]. IL-6 was assayed by a commercial ELISA kit (BMS213/2MST; Bender MedSystems, Austria); normal/reference maximum value: 14.1 pg/ml (mean: 1.3±3.2 pg/ml).

The presence of surface granulocyte activation markers: L-selectin, CD15 and lactoferrin was analysed by flow cytometry using an indirect immunofluorescent method; the process for labelling and hemolysis was started within 2 hours after blood collection. The protocol employed was the same as described before [18], except that a different CD15 monoclonal antibody was applied (Dako, 8.5 mg/l). (Other primary antibodies: a polyclonal anti-lactoferrin (200 mg/l, ICN), and a monoclonal anti-L-selectin (47 mg/l) (Dako); secondary antibodies: anti-rabbit (10 mg/l) or anti-mouse (20 mg/l) FITC-labelled antibodies (Dako); negative controls: normal rabbit serum or isotype-specific normal mouse Ig (Dako)). Immunolabelling of whole-blood EDTA samples was initiated within 1 h. Hemolysis was carried out after immunostaining (Lysing kit; Biodesign, USA). Investigations were carried out with a FACStar Plus Becton-Dickinson equipment; only the population of granulocytes was analysed from leukocytes. Granulocytes were gated on the basis of their characteristic forward- (FS) and side-scatter (SS) features; as they have relatively high side scatter, granulocytes could be easily distinguished from the two other, main leukocyte populations: i.e. from lymphocytes, and monocytes. (The elevated side scatter is related to the granularity of these leukocytes; the forward scatter is roughly proportional to the diameter of the cell.) As granulocytes constitute the predominant leukocyte fraction, we could easily delimit the area of their population with high FS in the flow cytometry procedure. Granulocytes with cell surface lactoferrin were identified as activated neutrophils, since lactoferrin is specific for neutrophils, and it can gain access to the cell surface only after degranulation triggered by cell activation [31]–[33]. 10 000 events/tube were recorded in granulocyte populations. All measurements were preceded by standard equipment calibrations, and each detection series started with setting the background intensity level using the proper negative controls. Ratios of labelled granulocytes (% of cells/granulocytes carrying/bearing labelled markers on cell surface) and mean fluorescence intensities (MFI, related to mean quantity of labelled molecules/cell) are shown.

### Statistical Analysis

For continuous variables, data are presented as mean ± standard deviation (SD) in Table 1 (upper part), and in the supplementary table (Table S1); in case of Figures, mean ± standard error of means (SEM) is used. General characteristics of the subject groups were compared by two-sample Student’s t-test. In case of categorical variables, number of subjects (n) and percentage (%) were given, and the groups were compared by Fisher’s exact test (Table 1, bottom). Distribution was checked by Kolmogorov-Smirnov statistics; in case of skewed distribution (ACTH), further analysis was performed on log-transformed data.

**Table 1:** Characteristics of the patient groups and routine blood analysis.

Variable	SAP (n = 21)	ACS (n = 20)	*P*
Age (y)	64.1±10.3	63.2±13.6	0.81
Total leukocyte count (G/l)	7.02±1.41	8.37±3.02	0.08
Neutrophil fraction (%)	62.62±7.46	65.83±8.18	0.20
Cholesterol (mmol/l)	4.34±1.28	5.01±1.10	0.11
HDL-cholesterol (mmol/l)	1.29±0.45	1.30±0.41	0.95
LDL-cholesterol (mmol/l)	2.36±1.04	3.13±1.04	0.043*
Triglycerides (mmol/l)	1.60±0.62	1.35±1.01	0.36
CK (U/l)	175.0±286.6	669.8±715.7	0.008*
Male gender (n, %)	13 (61.9)	14 (70.0)	0.74
Diabetes mellitus type II (n, %)	5 (23.8)	6 (30.0)	0.73
Hypertension (n, %)	16 (76.2)	16 (80.0)	1.00
Statins use (n, %)	16 (76.2)	15 (75.0)	1.00
Antihypertensives use (n, %)	21 (100)	17 (85.0)	0.11
Sedatives use (n, %)	7 (33.3)	7 (35.0)	1.00

SAP: stable angina pectoris, ACS: acute coronary syndrome patients.

Values of continuous variables are means ± SD, values of categorical variables are n (%).

Exceptions for sample numbers in lipid panel: total cholesterol, triglycerides (SAP: n = 19, ACS: n = 16); HDL-cholesterol (SAP: n = 18, ACS: n = 16); LDL-cholesterol (SAP: n = 18, ACS: n = 15). *p*<0.05 values (significant differences between the two groups) are denoted by asterisks (*).

In our analysis, there were two main factors: the time (pre-PCI, post-PCI and 1d-PCI) as within-group (“intragroup”) factor for repeated measurements and the two groups as between-subject (“intergroup”) factor for independent samples (Table S1). For over time comparisons (pre-PCI, post-PCI, and 1d-PCI samples), two-way repeated measurements ANOVA was used. The resulting *p* values were for overall intragroup *p*, between groups/intergroup *p*, and one for their interaction. Significant value of interaction allowed also between-group (SAP vs ACS) comparisons at each timepoint. When the *p* value of interaction was relatively low: 0.3>*p*>0.05, the presence of interaction was assumed to allow intergroup comparisons in these special statistical cases. Significancy of the separate intragroup *p* allowed further pairwise comparisons of the repeated measurements within each group. All the pairwise comparisons are modified t-tests, these and the separate intragroup *p*-s were calculated by the software based on estimated marginal means. The step-down Bonferroni adjustment was employed for multiple comparisons to keep the familywise type I error rate on α = 0.05 level. It is well known, that responses measured on the same subject are usually correlated; also, variances of repeated measures often change with time. We used a mixed model, where this correlation can be modelled. For our data, a general unstructured form was chosen for within-subject variance covariance matrix. Heterogeneity of group-variances was also taken into account. To examine the relationship between the stress hormones and selected inflammatory markers, Spearman’s coefficient of correlation and its significance were calculated (Table 2). Statistical analyses were carried out with SPSS (version 15, SPSS Inc., Chicago) and SAS (v9.1, SAS Institute, Cary, NC) softwares. All tests were two-sided, and *p*<0.05 was considered to be statistically significant.

**Table 2:** Correlations between stress hormones and selected inflammatory markers.

Correlations between simultaneousdata (patient group, time, parameter)	Surface lactoferrin-bearinggranulocytes (r)	Plasma lactoferrin (r)	Il-6 (r)
SAP - pre-PCI - Cortisol	0.051 (p = 0.827)	0.103 (p = 0.658)	−0.218 (p = 0.385)
SAP - pre-PCI - ACTH	−0.283 (p = 0.213)	−0.158 (p = 0.493)	−0.483* (p = 0.042)
ACS - pre-PCI - Cortisol	0.391 (p = 0.088)	0.499* (p = 0.025)	−0.135 (p = 0.581)
ACS - pre-PCI - ACTH	0.364 (p = 0.115)	0.398 (p = 0.082)	−0.298 (p = 0.215)
SAP - 1d-PCI - Cortisol	−0.192 (p = 0.404)	−0.015 (p = 0.950)	0.269 (p = 0.280)
SAP - 1d-PCI - ACTH	−0.601* (p = 0.004)	−0.435* (p = 0.049)	0.024 (p = 0.926)
ACS - 1d-PCI - Cortisol	0.281 (p = 0.244)	0.250 (p = 0.289)	0.254 (p = 0.293)
ACS - 1d-PCI - ACTH	−0.541* (p = 0.017)	−0.609* (p = 0.004)	−0.068 (p = 0.783)

SAP: stable angina pectoris, ACS: acute coronary syndrome patients; pre-PCI: directly before, and 1d-PCI: on the following day of PCI (stenting).

*r:* Spearman’s rank correlation coefficient; asterisks (*) denote significancy of correlations (*p*<0.05).

SAP: n = 21, ACS n = 20, except for IL-6 (ACS pre- and 1-d-PCI: n = 19) and for percentage of surface lactoferrin-bearing cells (in ACS patients 1-day after PCI: n = 19).

## Results

### General Characteristics of the Patient Groups and Routine Blood Data

Our study involved 21 stable angina pectoris and 20 acute coronary syndrome patients with a majority of males in both groups (Table 1). The only between-group differences found were: slightly increased LDL-cholesterol and markedly elevated CK activity in the ACS group. All other presented laboratory parameters were within the normal ranges.

### Stress Hormones (Cortisol, ACTH), Surface Activation Markers of Granulocytes (L-selectin, CD15, Lactoferrin) and Inflammatory Markers in Plasma (Lactoferrin, IL-6)

**Figure 1:**
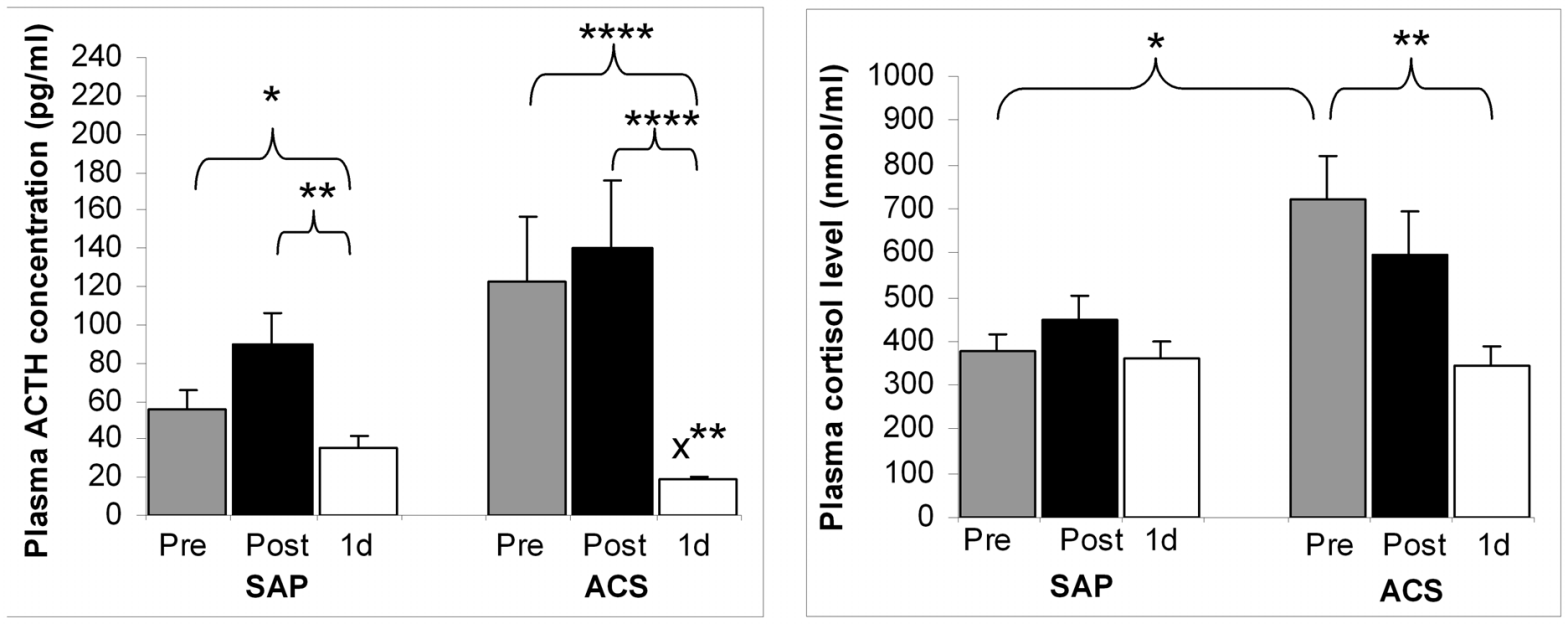
Plasma levels of ACTH and cortisol in CAD patients undergoing PCI. Blood samples were collected from stable angina pectoris (SAP) and from acute coronary syndrome (ACS) patients directly before (Pre), directly after (Post) and on the following day (1d) of PCI. Data are presented as mean ± SEM; in case of SAP, n = 21, and for ACS, n = 20. Significant intragroup differences: Pre/1d, Post/1d; p* <0.05, p** <0.01, p**** ≤0.0001; significant intergroup differences (SAP vs ACS) for ACTH: 1d, *p*x** = p** <0.01; for cortisol: Pre, p* <0.05.

On admittance to the clinic, ACS patients were in a stressed state, with raised plasma cortisol and grossly elevated ACTH concentration; these values decreased dramatically next day after PCI: to about 50% (cortisol: *p*<0.01), and to 15% (ACTH: *p* = 0.0001) (Fig. 1, Table S1). Cortisol values of SAP patients were in the normal range, and did not change before/after PCI. However, a substantial decline of the ACTH level was observed also in these patients the day after PCI (pre-PCI*/*1d-PCI: *p*<0.05, post-PCI*/*1d-PCI: *p*<0.01); interestingly, the 1d-ACTH value was significantly higher in the SAP group than in the ACS one (*p*<0.05). Significant (statistical) interactions could be demonstrated only in the case of the stress hormones (cortisol: *p*<0.001, ACTH: *p*<0.05) that allowed between-group comparisons.

**Figure 2:**
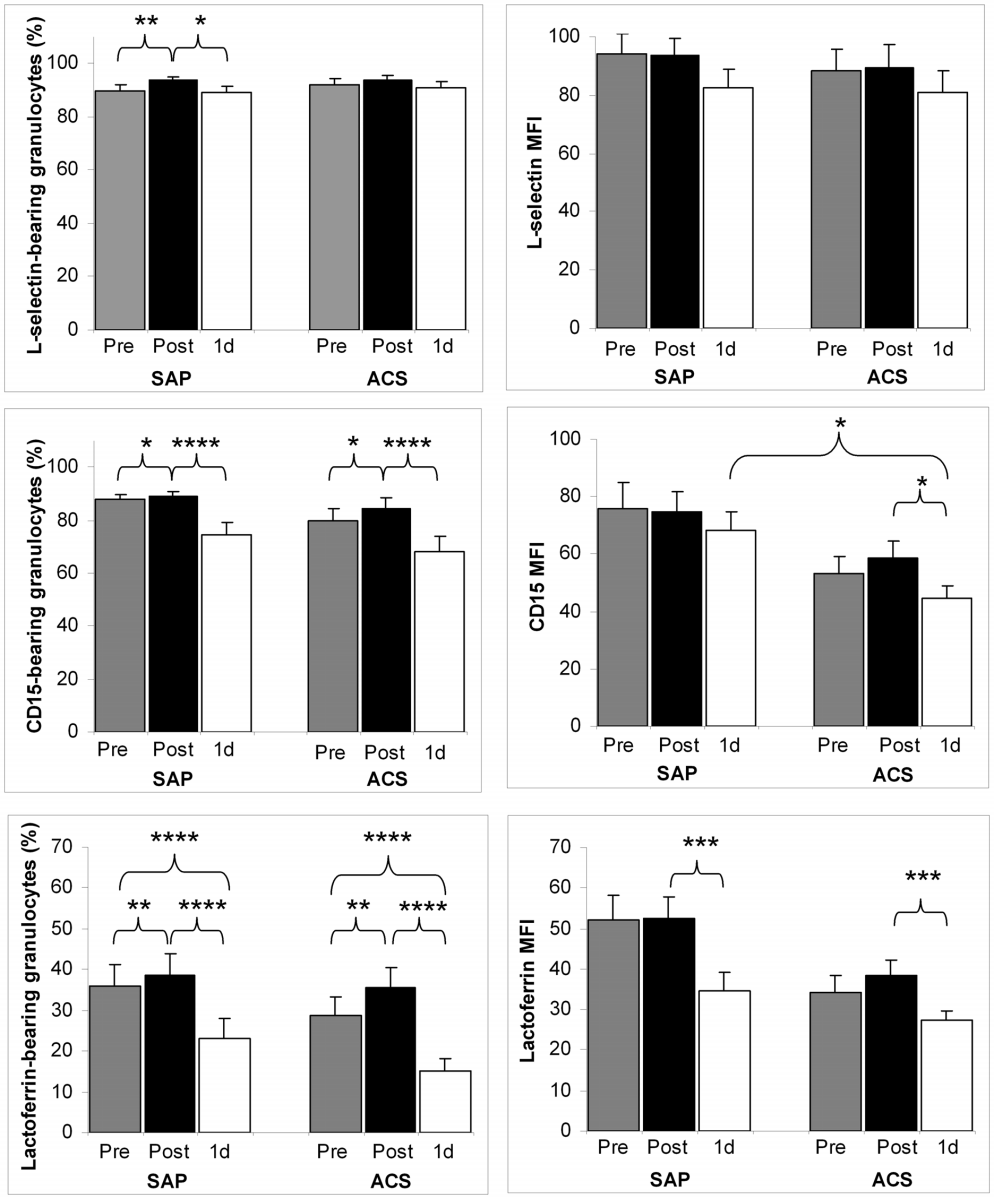
Flow cytometry analysis of granulocytes in CAD patients undergoing PCI. Percentages of marker-bearing granulocytes and mean fluorescence intensities (MFI) of granulocytes reflect the cell surface appearance of activation markers directly before, directly after and on the following day of PCI (Pre, Post, 1d) in patients with stable angina pectoris (SAP) or with acute coronary syndrome (ACS). Data are mean ± SEM; in case of SAP, n = 21, and for ACS, n = 20 (ACS exception: 1d values, n = 19). Significant differences: p* <0.05, p** <0.01, p*** <0.001, p**** ≤0.0001.

Considering cell surface appearances of L-selectin and CD15 (a selectin ligand), no (L-selectin MFI) or only mild changes could be seen in a time- and/or intervention-dependent manner (Fig. 2, Table S1). In the SAP group, proportion of L-selectin-carrier granulocytes slightly increased during PCI (*p*<0.01), and decreased next day, declining to about its initial value (*p*<0.05). Similarly to the previous parameter, no distinction could be made between groups considering the slightly rising (pre-PCI/post-PCI: *p*<0.05) and then mildly declining (post-PCI/1d-PCI: *p*<0.0001) ratios of CD15-bearing granulocytes. It is to be noted, that the 1d-PCI percentage of the CD15-bearing granulocytes was lower than the initial one (*p*<0.01) in both groups. Concerning CD15 MFI, it was reduced somewhat after PCI (*p*<0.05) in the ACS group, and difference between groups could be shown in 1d-PCI CD15 MFI (*p*<0.05).

**Figure 3:**
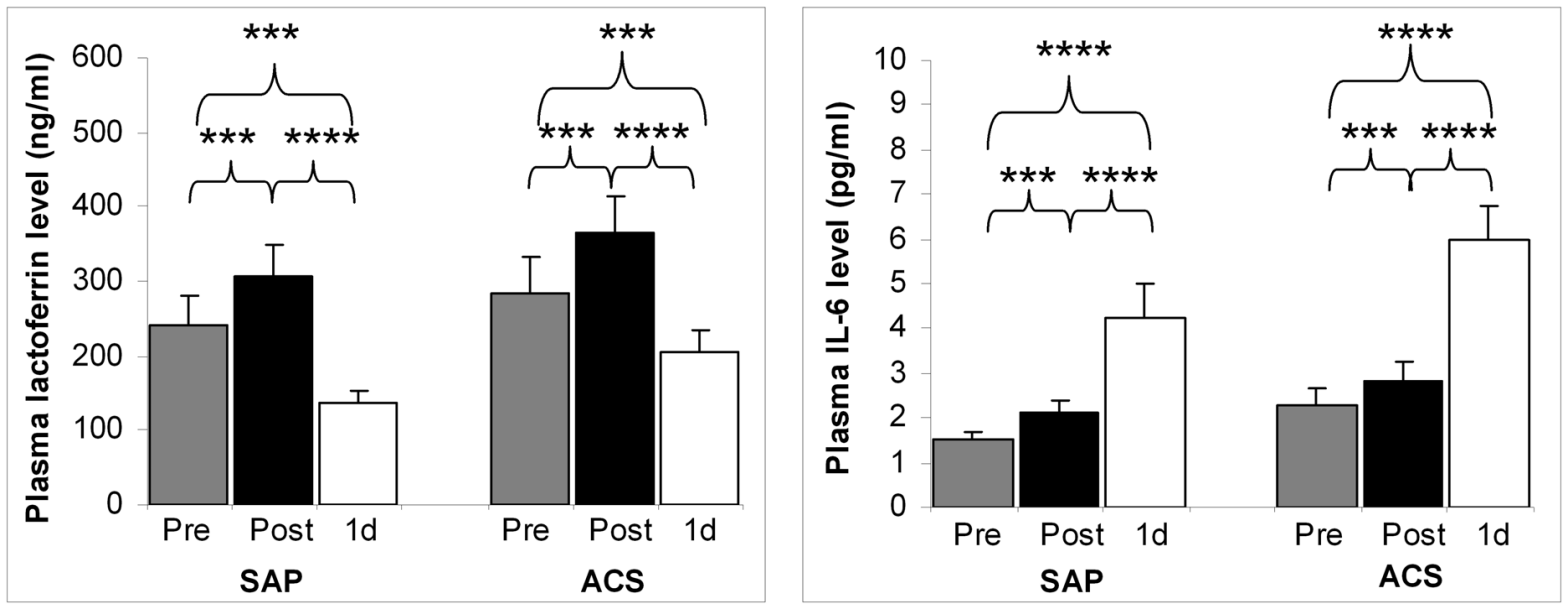
Plasma levels of lactoferrin and IL-6 in CAD patients undergoing PCI. Blood samples were collected from stable angina pectoris (SAP) and from acute coronary syndrome (ACS) patients directly before (Pre), directly after (Post) and on the following day (1d) of PCI. Data are presented as mean ± SEM; in case of SAP, n = 21 (IL-6: n = 18), and for ACS, n = 20 (IL-6: n = 19, except: Post: n = 18). Significant differences: p*** <0.001, p**** ≤0.0001.

More marked changes were seen in case of neutrophil-specific surface lactoferrin (Fig. 2, Table S1). The ratio of surface lactoferrin-bearing granulocytes approximately halved following PCI (post-PCI/1d-PCI: *p*<0.0001). The pre-PCI values were high relatively to the 1d-PCI ones, and elevated slightly during the intervention (*p*<0.01); interestingly, these 1d-PCI percentages were considerably lower than the pre-PCI ones (*p*<0.0001). Less substantial alterations were found in case of lactoferrin MFI after PCI (post-PCI/1d-PCI: *p*<0.001). Similarly to the proportion of surface lactoferrin-bearing granulocytes, plasma lactoferrin approximately halved till the next day after PCI (*p*<0.0001); these 1d-PCI values were significantly lower than the initial ones (*p*<0.001) (Fig. 3). In post-PCI samples, a moderate increase was observed (*p*<0.001). Pre- and post-PCI plasma lactoferrin concentrations were over the upper limit of the physiological range (200 ng/ml). Plasma IL-6 displayed a different pattern of changes: a mild increase immediately after intervention was followed by an about 2-fold elevation (pre-PCI/post-PCI: *p*<0.001, post-PCI/1d-PCI: *p*<0.0001).

### Correlations between Stress Hormones (Cortisol, ACTH) and Selected Markers: Percentage of Surface Lactoferrin-bearing Neutrophils, Plasma Lactoferrin, IL-6

As the most substantial alterations were found in the ratio of surface lactoferrin-bearing cells, plasma lactoferrin and IL-6, correlations among these inflammatory markers and the assayed stress hormones were tested (Table 2). In 1d-PCI samples of both groups, plasma ACTH showed inverse associations with the ratio of lactoferrin-bearing neutrophils (SAP: *r* = −0.601, *p*<0.005; ACS: *r* = −0.541, *p*<0.05) and with plasma lactoferrin (SAP: *r* = −0.435, *p*<0.05; ACS: *r* = −0.609, *p*<0.005). In addition, ACTH correlated negatively with IL-6 in post-PCI samples of both groups (SAP: *r* = −0.487, *p*<0.05; ACS: *r* = −0.503, *p*<0.05), and in the pre-PCI sample of the SAP group (*r* = −0.483, *p*<0.05). In contrast, cortisol was positively associated with plasma lactoferrin in ACS patients at the start (*r = *0.499, *p*<0.05).

## Discussion

Our results suggest that pre- and post-PCI states could be associated with increased percentage of activated/degranulated neutrophils releasing lactoferrin. In the highly stressed ACS patients with elevated HPA axis activity, plasma lactoferrin correlated with plasma cortisol before PCI. The following day after PCI, the ratio of surface lactoferrin-carrier granulocytes and plasma lactoferrin decreased in both groups that could reflect the negative immune-modulatory effect of ACTH (as suggested by negative correlations).

Our ACS patients appeared to be in an extremely stressed state before PCI indicated by highly elevated plasma cortisol, which was substantially reduced on the day after PCI. The non-reactive, unchanged cortisol level of SAP patients could be due to a previously overloaded, exhausted HPA axis [34]. In ACS, the overactivated HPA axis switched to a much decreased, normal functioning state during the hours after stenting; the final low plasma ACTH may reflect a gross relief following PCI. In SAP group, the somewhat decreased 1d-PCI ACTH value could mark a more relaxed state relatively to the initial one. As the peaks of ACTH and cortisol concentrations in blood are in the early morning period/before 6 a.m./, the (statistically) significant decreases in plasma ACTH and cortisol in the 1d-PCI samples (collected routinely between 8–9 a.m.) are most probably not related to the diurnal fluctuation.

L-selectin is constitutively expressed in the leukocyte plasma membrane. Activation is thought to lead mostly to its cleavage/shedding from the cell surface, like in unstable angina pectoris [28]; however, increased cell surface appearance could also accompany activation, e.g. after mechanical trauma [35]. In the ratio of L-selectin-bearing granulocytes of SAP patients, the slight increase during PCI followed by a moderate decrease is not remarkable (although statistically significant); this might indicate homeostatic alterations in these activated leukocytes (shedding balanced by reappearance).

CD15 (Lewis-X) is a tetrasaccharide ligand (to selectins) present on all myeloid cells; in neutrophils, it can be mobilized from azurophilic/primary granules [36]. The statistically significant, slight increase in post-PCI ratio of CD15-bearing cells is not considerable; it could reflect slight granulocyte activation during the intervention in both patient groups. The moderate down-regulation on the following day, however, indicates a less activated state, similarly to the granulocytes of anxious patients in a relatively relaxed condition in our previous study [18].

Lactoferrin represents a specific activation marker of neutrophils, which can be bound to cell surface and can gain access to the circulation only after its release from secondary granules upon cell activation [31]–[33]. It is worth noting, that ^125^I-labelled lactoferrin was shown to be rapidly cleared from the circulation, with all lactoferrin being removed within 7 hours after injection in humans [37]. The pronounced reductions in cell surface and plasma lactoferrin parameters mark a less activated state of neutrophils a day after PCI, independently of patient group. Directly after intervention, a mild, not remarkable increase was observed in the proportion of marker-carrier neutrophils in both patient groups, similarly to the case of CD15. These intervention-related moderate changes could be observed also in plasma lactoferrin, which reflect limited neutrophil activation. Gach et al. described a steep rise of plasma lactoferrin in coronary blood immediately after PCI in patients with unstable angina, that was reversed in 6 hours [28]. In our study, the pre-PCI values of surface lactoferrin-bearing granulocytes were also high (>25%), indicating enhanced percentage of activated neutrophils in CAD. Our results on elevated pre-PCI plasma lactoferrin in both groups correspond to those of Videm et al., who proposed raised plasma lactoferrin as an excellent marker for significant atherosclerotic coronary stenosis and found it to be more reliable, than plasma myeloperoxidase (another neutrophil activation marker) [10]. Even more, increased baseline plasma lactoferrin was suggested to predict long-term risk for fatal CAD in patients with newly diagnosed diabetes [38]. As plasma lactoferrin and the percentage of surface lactoferrin-carrier granulocytes showed similar, marked changes in our patient groups, both may be used as „neutrophil activity sensors” in CAD patients. From the practical point of view, however, plasma lactoferrin assays appear to be more favourable; (less expensive tests which probably could be automated).

Onset of acute phase reaction was seen as a rising plasma IL-6, particularly enhanced a day after PCI, when activation level of neutrophils was already reduced. Similarly, a decrease of plasma lactoferrin and myeloperoxidase and increase of IL-6 were observed during 6–12 hours after stenting in unstable angina [28]. Caixeta et al. found a statistically significant, but moderate increase of IL-6 level at 6 h after PCI both in unstable and in stable angina patients followed by a decline; however, this study may have lost the peak at 24 h – as suggested [27]. It is worth noting, that psychological stress was found to have strong relationship with circulating level of IL-6 in treated HIV-infected individuals [39].

Our principal finding is that correlations could exist among the neutrophil activation state and plasma cortisol and/or ACTH in CAD patients. It is plausible to suppose that ACTH could be a suppressor of neutrophil activation/degranulation: its plasma level was inversely associated with the ratio of surface lactoferrin-bearing granulocytes and plasma lactoferrin in both groups a day after PCI. This is in agreement with the concept of tonal inhibition of immune/inflammatory responsiveness by ACTH [40]. Correlation of cortisol with plasma lactoferrin before PCI in ACS seems to be controversial, as cortisol and its analogs are known as anti-inflammatory agents and immune suppressors. However, glucocorticoids were also reported to activate human neutrophils in vitro; and the relatively high ratio of α to β type of glucocorticoid receptors is suggested to be responsible for neutrophil insensitivity to apoptosis induction by these hormones [21], [41]. Furthermore, as morning plasma cortisol levels were found to correlate with the severity of coronary atherosclerosis in US Air Force aircrew members and in patients with suspected CAD, cortisol may be considered as a proatherogenic stress hormone [22]–[23]. This notion is supported by some investigations finding that selective inhibitors of the enzyme specifically involved in cortisol synthesis (11β-hydroxysteroid dehydrogenase) could be useful in prevention of atherosclerosis [42]–[43].

Neutrophil granulocytes could be activated as part of an ancient adaptation/survival process in case of danger/stress [44]. Stimulation of neutrophils could be involved in the initiation of atherosclerosis in a severalfold way. Hypochlorous acid (HOCl) produced by neutrophil-specific myeloperoxidase was reported to play a critical role in the production of oxidized LDL, that further stimulates neutrophils leading to activation of respiratory burst (production of reactive oxygen species) and to degranulation (release of enzymes, some adhesion receptors, lactoferrin etc.). Interestingly, HDL apolipoprotein AI and vitamin C could be protective [45]–[46]. Additionally, alfa-defensins (neutrophil peptides) could be also involved in the cardiovascular inflammatory reaction [47].

There are some limitations to be discussed. First, the exact mechanisms in the background of plasma ACTH/cortisol correlations with the neutrophil activation state (inflammatory state) could not be clarified in this clinical study. Second, several characteristics of the patient groups might confound the described changes: e.g. life style factors, age, sex, and pre-existent illnesses. It is to be noted that metabolic syndrome could affect ACTH levels; in addition, this syndrome is widely known to be often accompanied by CAD [48]–[49]. However, our patient groups functioned as auto-controlled statistical units (i.e. repeated measurements within-group, over-time comparisons), therefore, the confounding factors listed above had probably limited influence. The relatively small sample size must be also considered; still, the high levels of statistical significances support the validity of our results.

Despite the apparent limitations, our study offers interesting insights into the dynamically changing neutrophil behaviour in CAD patients undergoing stenting. We showed that neutrophil activation could be enhanced already before stenting, and that neutrophils could be less activated next day after PCI - after a mild, transitory increase in their activation state directly after stenting. Plasma lactoferrin appeared to be a sensitive and valuable marker of neutrophil activation, which deserves detailed methodological studies to evaluate it as a risk marker for CAD. To our best knowledge, we are the first to find correlations between plasma ACTH, cortisol and neutrophil activation/degranulation state in CAD patients before and/or after stenting; further studies are required to elucidate the underlying mechanisms.

## Supporting Information

Table S1 **Statistical analysis of stress hormone and inflammatory marker values in SAP and ACS patients (mean ± SD,** ***p-values*** **).** The table contains the results of the detailed statistical analysis: significancy values of intra- and intergroup differences (*p* values of intragroup/within-group comparisons for repeated measurements: pre-PCI vs post-PCI, post-PCI vs 1d-PCI, and pre-PCI vs 1d-PCI; separate intragroup *p* values for SAP or ACS; overall intragroup *p* values; and intergroup/between groups *p* values). Significancy of the separate intragroup *p* was a prerequisite for pairwise comparisons of the repeated measurements within each group. The *p* value of interaction is also presented; its significancy or its relatively low value (0.3>*p*>0.05) allowed also between-group (SAP vs ACS) comparisons at each timepoint.
(DOC)
