# Supplementary material for: ACTH- and Cortisol-Associated Neutrophil Modulation in Coronary Artery Disease Patients Undergoing Stent Implantation
Source: PLoS One. 2013 Aug 14;8(8):e71902. doi: 10.1371/journal.pone.0071902 (PMC3743772; doi:10.1371/journal.pone.0071902)
Supplement: Table S1 — Statistical analysis of stress hormone and inflammatory marker values in SAP and ACS patients (mean ± SD, p-values ). The table contains the results of the detailed statistical analysis: significancy values of intra- and intergroup differences (p values of intragroup/within-group comparisons for repeated measurements: pre-PCI vs post-PCI, post-PCI vs 1d-PCI, and pre-PCI vs 1d-PCI; separate intragroup p values for SAP or ACS; overall intragroup p values; and intergroup/between groups p values). Significancy of the separate intragroup p was a prerequisite for pairwise comparisons of the repeated measurements within each group. The p value of interaction is also presented; its significancy or its relatively low value (0.3>p>0.05) allowed also between-group (SAP vs ACS) comparisons at each timepoint. (DOC) [file pone.0071902.s001.doc]

**Table S1.** Statistical analysis of stress hormone and inflammatory marker values in SAP and ACS patients (mean ± SD, *p-values*)

| Parameters | Sample results, *significancy of intra- and intergroup differences^a^* | | | | *ANOVA analysis^c^* | | | |  |
| --- | --- | --- | --- | --- | --- | --- | --- | --- | --- |
|  | Pre-PCI (1) | Post-PCI (2) | 1d-PCI (3) | *Separate intragroup p^b^* |  | *Overall intragroup p* | *Between groups*  *(intergroup p)* | *Interaction* | |
| Cortisol (nmol/l) |  |  |  |  |  | *0.001* | *0.0499* | *0.0006* | |
| SAP | 375.81±173.66 | 450.00±242.96 | 360.43±183.71 | *0.706* |  |  |  |  | |
| ACS | 722.20±440.56 | 595.80±440.84 | 342.40±193.99 | *0.006** |  |  |  |  | |
| *ACS intragroup p^b^* | *1 vs 2: 0.184* | *2 vs 3: 0.060* | *1 vs 3: 0.002** |  |  |  |  |  | |
| *SAP vs ACS p^b^* | *0.018** | *0.610* | *0.762* |  |  |  |  |  | |
|  |  |  |  |  |  |  |  |  | |
| ACTH (pg/ml) *^d^* |  |  |  |  |  | *0.0001* | *0.940* | *0.023* | |
| SAP | 55.56±46.15 | 89.80±76.65 | 35.64±25.58 | *0.014** |  |  |  |  | |
| *SAP intragroup p^b^* | *1 vs 2:* 0.102 | *2 vs 3:* 0.004* | *1 vs 3:* 0.03* |  |  |  |  |  | |
| ACS | 122.60±154.41 | 140.00±158.03 | 18.79±8.44 | *0.0001** |  |  |  |  | |
| *ACS intragroup p^b^* | *1 vs 2: 0.507* | *2 vs 3: 0.0001** | *1 vs 3: 0.0001** |  |  |  |  |  | |
| *SAP vs ACS p^b^* | *0.544* | *0.544* | *0.007** |  |  |  |  |  | |
| L-selectin MFI |  |  |  |  |  | *0.092* | *0.735* | *0.732* | |
| SAP | 94.01±31.64 | 93.64±27.01 | 82.39±29.35 |  |  |  |  |  | |
| ACS | 88.10±33.36 | 89.64±34.89 | 80.79±32.36 |  |  |  |  |  | |
| L-selectin-bearing granulocytes (%) |  |  |  |  |  | *0.0002* | *0.613* | *0.147* | |
| SAP | 90.00±8.92 | 93.71±4.65 | 88.94±10.50 | *0.001** |  |  |  |  | |
| *SAP intragroup p* | *1 vs 2: 0.001** | *2 vs 3: 0.021** | *1 vs 3: 0.557* |  |  |  |  |  | |
| ACS | 92.33±8.34 | 93.53±8.00 | 90.62±11.59 | *0.302* |  |  |  |  | |
| *SAP vs ACS p* | *1.000* | *1.000* | *1.000* |  |  |  |  |  | |
|  |  |  |  |  |  |  |  |  | |
| CD15 MFI |  |  |  |  |  | *0.048* | *0.013* | *0.248* | |
| SAP | 75.78±42.52 | 74.60±32.24 | 68.19±30.47 | *0.789* |  |  |  |  | |
| ACS | 53.38±25.54 | 58.61±27.25 | 44.89±18.35 | *0.032** |  |  |  |  | |
| *ACS intragroup p* | *1 vs 2: 0.540* | *2 vs 3: 0.032** | *3 vs 1: 0.17* |  |  |  |  |  | |
| *SAP vs ACS p* | *0.332* | *0.332* | *0.032** |  |  |  |  |  | |
| CD15-bearing  granulocytes (%) |  |  |  |  |  | *<0.0001* | *0.199* | *0.759* | |
| SAP | 87.88±8.26 | 89.25±8.29 | 74.59±22.12 |  |  |  |  |  | |
| ACS | 79.86±19.41 | 84.16±18.16 | 68.33±24.46 |  |  |  |  |  | |
| *SAP or ACS intragroup p* | *1 vs 2: 0.018** | *2 vs 3: <0.0001** | *3 vs 1: 0.002** |  |  |  |  |  | |
|  |  |  |  |  |  |  |  |  | |
| Lactoferrin MFI |  |  |  |  |  | *0.001* | *0.017* | *0.436* | |
| SAP | 52.06±28.19 | 52.38±25.25 | 34.48±20.97 |  |  |  |  |  | |
| ACS | 34.25±18.65 | 38.54±16.51 | 27.45±9.69 |  |  |  |  |  | |
| *SAP or ACS intragroup p* | *1 vs 2: 0.397* | *2 vs 3: 0.0006** | *3 vs 1: 0.01** |  |  |  |  |  | |
| Lactoferrin-bearing granulocytes (%) |  |  |  |  |  | *<0.0001* | *0.322* | *0.376* | |
| SAP | 35.80±25.14 | 38.62±24.47 | 23.26±21.23 |  |  |  |  |  | |
| ACS | 28.57±20.31 | 35.74±21.15 | 15.07±13.98 |  |  |  |  |  | |
| *SAP or ACS intragroup p* | *1 vs 2: 0.008** | *2 vs 3: <0.0001** | *3 vs 1: <0.0001** |  |  |  |  |  | |
|  |  |  |  |  |  |  |  |  | |
| Plasma lactoferrin  (ng/ml) |  |  |  |  |  | *<0.0001* | *0.267* | *0.826* | |
| SAP | 241.86±178.75 | 306.32±193.49 | 137.69±71.61 |  |  |  |  |  | |
| ACS | 282.10±220.1 | 365.72±220.47 | 205.33±124.61 |  |  |  |  |  | |
| *SAP or ACS intragroup p* | *1 vs 2: 0.0008** | *2 vs 3: <0.0001** | *3 vs 1: 0.0008** |  |  |  |  |  | |
|  |  |  |  |  |  |  |  |  | |
| Plasma IL-6  (pg/ml) |  |  |  |  |  | *<0.0001* | *0.057* | *0.482* | |
| SAP | 1.52±0.68 | 2.11±1.16 | 4.26±3.13 |  |  |  |  |  | |
| ACS | 2.31±1.48 | 2.80±1.88 | 5.99±3.22 |  |  |  |  |  | |
| *SAP or ACS intragroup p* | *1 vs 2: 0.0009** | *2 vs 3: <0.0001** | *3 vs 1: <0.0001** |  |  |  |  |  | |

Percentages of surface marker-bearing granulocytes and mean fluorescence intensities (MFI) of granulocytes, additionally, plasma levels of lactoferrin and IL-6 in patients with stable angina pectoris (SAP) or with acute coronary syndrome (ACS); directly before, directly after and on the following day of PCI (Pre, Post, 1d). Data are mean ± SD; in case of SAP, n = 21 (IL-6: n = 18), and for ACS, n = 20 (1d granulocyte values, n = 19; IL-6: n = 19, except: Post: n = 18).

*^a^*  the intragroup and intergroup differences were calculated by special contrasts, based on estimated marginal means; after the correction by step-down Bonferroni method; significant intragroup differences (*p* < 0.05 values) are denoted by asterisks (*); *^b^* in case of the presence of interaction (between groups), *p* values are given after the correction by step-down Bonferroni method; *^c^* *p* values of the two-way ANOVA; *^d^* ACTH statistical analysis: log transformated data (ANOVA was performed on the logarithm of data).
